# Supplementary material for: Analysis of PPARγ Signaling Activity in Psoriasis
Source: Int J Mol Sci. 2021 Aug 10;22(16):8603. doi: 10.3390/ijms22168603 (PMC8395241; doi:10.3390/ijms22168603)
Supplement: Supplementary file 1 [file ijms-22-08603-s001.zip › Supplemental materials_Analysis of PPARg signaling activity in psoriasis/Pathway models/Models images and html files/Interleukin-17 and interleukin-22 signaling in psoriasis/5883.html]

Ras GTPase


# Functional Class Ras GTPase

|  |  |
| --- | --- |
| URN | urn:agi-aopfc:0000465 |
| Total Entities | 19 |
| Connectivity | 6357 |
| Name | Ras GTPase |
| Description | RAS oncogene homolog |
| ObjectType | Functional Class |

---

|  |  |
| --- | --- |
| ChildConcepts | HRAS |
|  | ERAS |
|  | KRAS |
|  | NRAS |
|  | Ral GTPase |
|  | RIT2 |
|  | RIT1 |
|  | RRAS |
|  | RASL10A |
|  | IFT27 |
|  | RRAS2 |
|  | MRAS |
|  | RASL12 |
|  | RAB6B |
|  | RASL11B |
|  | RERGL |
|  | RERG |
|  | RASL10B |
|  | RASL11A |

---

|  |  |
| --- | --- |
| Pathway | Alzheimer's Disease |
|  | Osteoarthritis |
|  | Skin Aging |
|  | mTOR Signaling |
|  | mTOR Signaling Hyperfunction |
|  | mTOR Signaling Activation by Taste Sensor Receptors (Rodent Model) |
|  | Insulin Influence on Protein Synthesis |
|  | Insulin Influence on Glycogenesis |
|  | Dopamine Receptors Signaling |
|  | Serotonin Receptors Signaling |
|  | Catecholamines Secretion from Adrenal Gland |
|  | Thyroid Hormones Common non-Genomic Effects in Hyperthyroidism |
|  | CRH Secretion Regulation |
|  | Prolactin Production |
|  | Thrombopoietin Receptors Signaling in Platelet Maturation |
|  | Mineralcorticoids Secretion Regulation |
|  | T-Cell Dependent B-Cell Activation |
|  | Natural Killer Cell Activation through C-Type Lectin-like Receptors |
|  | Natural Killer Cell Activation through ITAM-Containing Receptors |
|  | Natural Killer Cell Activation through Integrins and non-ITAM-Containing Receptors |
|  | T-Cell Maturation (Hypothesis) |
|  | Natural Killer Cell Activation |
|  | CD8+ T-Cell Activation |
|  | Th1-Cell Differentiation |
|  | Treg-Cell Differentiation |
|  | Th2-Cell Differentiation |
|  | T-Cell Receptor Signaling |
|  | CXC Chemokine Receptor Signaling |
|  | CC Chemokine Receptor Signaling |
|  | Plasmin Effects in Inflammation |
|  | Platelet Activation via Adhesion Molecules |
|  | Proplatelet Maturation |
|  | Platelet Activation via GPCR Signaling |
|  | Toll-like Receptor Independent Sterile Inflammation |
|  | MC1R in anti-Inflammatory Signaling |
|  | Leukotriene Effect on Airway Smooth Muscle Response |
|  | Basophil Activation |
|  | Eosinophil Survival by Cytokine Signaling |
|  | Mast-Cell Activation via IgE Signaling |
|  | Mast-Cells De Novo Synthesized Mediators via IgE Independent Signaling |
|  | CD16/CD14 Proinflammatory Monocyte Activation |
|  | Macrophage M2-Related Phagocytosis |
|  | anti-Inflammatory Function of Macrophage M2 Lineage |
|  | Neutrophil Recruitment and Priming |
|  | Neutrophil Degranulation via FPR1 Signaling |
|  | CR3 Mediated Phagocytosis in Neutrophils and Macrophages |
|  | Neutrophil Chemotaxis |
|  | Negative Acute Phase Proteins Synthesis |
|  | Positive Acute Phase Proteins Synthesis |
|  | Vascular Smooth Muscle Cell/Pericyte Migration and Proliferation |
|  | Vascular Endothelial Cell Activation by Blood Coagulation Factors |
|  | Vascular Endothelial Cell Activation by Growth Factors |
|  | Nociception Expression Targets Signaling |
|  | Vascular Motility |
|  | NPY1R -> CRH/POMC Production |
|  | NTSR1 Expression Targets |
|  | EDNRA/B -> Vascular Motility |
|  | ADRA1 -> Vasoconstriction |
|  | ADRA1A -> IL6 Production |
|  | ADRA2C/ADRB2 -> Vasoconstriction |
|  | ADRA2C/ADRB2 -> Synaptic Endocytosis |
|  | ADRA1 -> Prostaglandin Generation |
|  | BDKRB1/2 -> Prostaglandin Generation |
|  | CNR1/2 -> IL1B/2/4/6/10 Production |
|  | DRD2 Expression Targets |
|  | GALR1/2/3 -> POMC/NPY Production |
|  | HRH2/4 -> IL6/10 Production |
|  | CHRM1 -> IL2 Production |
|  | CHRM1/2/3 -> Vascular Motility |
|  | OPRK -> Pain Perception |
|  | PTGIR -> IL6 Production |
|  | PTGER1/4 -> Vascular Motility |
|  | HTR1 -> IL6 Production |
|  | HTR5 -> TNF Production |
|  | HTR1 -> Membrane Transport |
|  | IL6 Expression Targets -> Nociception |
|  | P2RY11/13/14 -> IL8/10 Production |
|  | Luteinizing Hormone in Oocyte Meiotic Resumption |
|  | Vitamine D Deficite and Dentin Formation |
|  | non-Genomic Rapid Actions of Vitamin D in Vitamin D Biology |
|  | G0/G1 Phase Transition |
|  | Th1-Cells Activation and Proliferation in Atherosclerosis |
|  | Cardiomyocyte Hypertrophy |
|  | Hypertrophic Cardiomyopathy |
|  | Endothelin Signaling in Arterial Hypertension |
|  | Aldosterone Synthesis Acute Regulation |
|  | Aldosterone Synthesis Chronic Regulation |
|  | TRPM4/6/7/8 Signaling in Arterial Hypertension (Hypothesis) |
|  | Smooth Muscle Cell Dysfunction in Pulmonary Hypertension |
|  | Contraction Due Vasospasm |
|  | Sebocyte Proliferation in Acne Vulgaris |
|  | Inflammatory Reaction in Acne Vulgaris |
|  | Hair Follicle Keratinocyte Apoptosis |
|  | Androgens in Sebocyte Maturation |
|  | Atopic Dermatitis Onset |
|  | Mast-Cells Activation in Atopic Dermatitis |
|  | MITF as a Regulator of Melanoma Cell Development |
|  | IFN-gamma/TNF-alpha Mediated Cell Proliferation in Psoriasis |
|  | IL22 Induces Keratinocyte Proliferation in Psoriasis |
|  | Th0-Cell Aberrant Activation in Systemic Lupus Erythematosus |
|  | Th1-Cell Function in Systemic Lupus Erythematosus |
|  | Th2-Cell Function in Systemic Lupus Erythematosus |
|  | Acute Cytotoxic CD8+T-Cell Response against Melanocytes |
|  | Vitiligo |
|  | T-Cell Cytotoxic Response against Melanocytes in Vitiligo |
|  | Apoptosis of Melanocyte in Vitiligo |
|  | Humoral Immunity in Vitiligo |
|  | CD8+ T-Cell Response to Self-Determinants in Diabetes |
|  | Treg-Cell Function in Diabetes Mellitus Type 1 |
|  | Natural Killer T-Cell Roles in Diabetes Mellitus Type 1 |
|  | non-Suppressive Treg-Cell in Diabetes Mellitus Type 1 |
|  | TLR2 Signaling in Treg-Cell in Type 1 Diabetes (Animal Model) |
|  | Natural Killer Cell in Diabetes Mellitus Type 1 |
|  | Peripheral Tolerance to Autoantigens Recession in Diabetes Mellitus Type 1 |
|  | Treg-Cell Activation in Diabetes Mellitus |
|  | Donohue Syndrome Progression (Hypothesis) |
|  | Tubular Cell Dysfunction in Progressive Diabetic Nephropathy |
|  | Endothelial Cell Dysfunction in Progressive Diabetic Nephropathy |
|  | Mesangial Cell Dysfunction in Diabetic Nephropathy |
|  | Proliferative Diabetic Retinopathy |
|  | Adiponectin Synthesis Declines in Insulin Resistance |
|  | Insulin Resistance in Hepatocytes |
|  | Hepatic Stellate Cells in non-Alcoholic Fatty Liver Disease |
|  | G-Proteins Signaling in Insulin Secretion in beta-Cell |
|  | Olanzapine Effect on Insulin Secretion in Diabetes Mellitus |
|  | Ghrelin Influence on Insulin Secretion (Hypothesis) |
|  | FOXO1 and SREBP-1C Role in beta-Cell Suppression (Rodent Model) |
|  | Hyperparathyroidism, Neonatal Severe |
|  | RET1 Mutation in Hyperparathyroidism |
|  | Hypothyrodism Associated Hyperprolactinemia |
|  | DRD2 Pharmacological Inhibition in Hyperprolactinemia |
|  | Ethanol Induced Efects in Hyperprolactinemia |
|  | Bone Remodeling in Hyperthyroidism |
|  | Cardiovascular Effects in Hyperthyroidism |
|  | GNRH Signaling Impairment in Hypogonadotrophic Hypogonadism |
|  | CD4+T-Cell Response in Celiac Disease |
|  | CD8+ T-Cell Response in Celiac Disease |
|  | Celiac Disease |
|  | Metastatic Colorectal Cancer |
|  | EDN, NRG, NRTN, and GDNF/RET Signaling in Hirschsprung Disease |
|  | Th2-Cell Function in Ulcerative Colitis |
|  | T-Cell Cytotoxic Response in Ulcerative Colitis |
|  | RARA Signaling in Acute Myeloid Leukemia (M3) |
|  | B-Cell Acute Lymphoblastic Leukemia |
|  | B-Cell Chronic Lymphocytic Leukemia |
|  | Proteins Involved in Chronic Myeloid Leukemia |
|  | Follicular Lymphoma |
|  | Hodgkin Lymphoma |
|  | IL6/IGF1/VEGFA Signaling in Multiple Myeloma |
|  | Proteins Involved in Multiple Myeloma |
|  | T-Cell Acute Lymphoblastic Leukemia |
|  | TRPM7/8 in Pancreatic Adenocarcinoma |
|  | Airway Smooth Muscle Cell High Rate Proliferation |
|  | IgE Induces Airway Smooth Muscle Cell Proliferation |
|  | Goblet-Cell Related Mucus Secretion in Asthma |
|  | Basophil Activation in Asthma |
|  | Mast-Cell Activation in Asthma |
|  | Antigen-Presenting Cell Role in Asthma |
|  | Th2-Cell Response in Asthma |
|  | Mucin Production in Goblet Airway Epithelial Cells |
|  | CFTR Facilitates Oxidative Stress in Airway Epithelium in CF |
|  | TNF and IL1B Induce Metalloproteinase Synthesis in Osteoarthritis |
|  | Steroidogenesis Impairement in Polycystic Ovary Syndrome |
|  | Ovulation Block |
|  | Polycystic Ovary Syndrome |
|  | FSH Action in Polycystic Ovary Syndrome |
|  | PTPN22 Role in Rheumatoid Arthritis |
|  | Synovial Fibroblast Proliferation in Rheumatoid Arthritis |
|  | B-Cells Function in Systemic Scleroderma |
|  | Th2-Cells Function in Systemic Scleroderma |
|  | Skin Fibrosis |
|  | Dentin Formation Disruption |
|  | CD8+ T-Cell Action Impairment in HIV Type 1 Infection |
|  | CD4+ T-Cell Function Decline in HIV |
|  | CD4+ T-Cell Death in HIV |
|  | Estrogen Effects on Pregnancy Gingivitis |
|  | IL1 Receptor Antagonist (IL1RN) Deficiency (DIRA) |
|  | Cowden Syndrome |
|  | Gluten Impact on Neuronal System (Hypothesis) |
|  | Myostatin in Skeletal Muscles in Muscular Dystrophies |
|  | IL6 Promotes Inflammation in Muscular Dystrophies |
|  | Muscular Dystrophies |
|  | Dystrophin Glycoprotein Complex Signaling in Muscular Dystrophies |
|  | Osteoblast Function Decline in Osteoporosis |
|  | Proteins with Altered Expression in Rheumatoid Arthritis |
|  | APP and Glutamate Signaling Cause Neuronal Dysfunction in Alzheimer Disease |
|  | BDNF Upregulation Triggered by Seizures |
|  | Apoptosis and Immediate Early Gene Activation in Epileptiform Disorders |
|  | Synaptic Exocytosis in Dementia |
|  | Glioblastoma, Primary |
|  | Glioblastoma, Secondary |
|  | Astrocytoma |
|  | Glioma Invasion Signaling |
|  | Ependymoma |
|  | Growth Factor Signaling in Neuroblastoma |
|  | Neuroblastoma |
|  | Microglia and Motor Neuron Interaction Dysregulation |
|  | ERBB/VEGFR/Akt Signaling in Breast Cancer |
|  | IGF1R/AKT Signaling in Breast Cancer |
|  | ESR1/ERBB Positive Luminal Breast Cancer |
|  | Breast Cancer |
|  | Angiogenesis in Endometriosis |
|  | Coagulation Factors and PAR1/2 Receptors (F2R/F2RL1) in Endometriosis |
|  | Leptin Role in Endometriosis |
|  | MIF Role in Cholesteatoma |
|  | Subepithelial Fibroblasts in Middle Ear Cholesteatoma |
|  | Cholesteatoma |
|  | HPV E5 Regulates EDNRA and EGFR Signaling Pathway in Keratinocytes |
|  | Prostate Cancer |
|  | TRPM8 Effects in Prostate Cancer (Hypothesis) |
|  | Steroids Induced Cataract |
|  | VEGFA in Neovascular Glaucoma |
|  | CDKN2B-AS1 in Epigenetic Regulation of Glaucoma |
|  | Glutamate Mediated Excitotoxicity in Glaucoma |
|  | PGF2-alpha Effect on Treatment |
|  | Cytokines Trigger Otitis Media |
|  | Alveolar Macrophage Negative Regulation Declined in COPD |
|  | Alveolar Epithelial Cell Dysfunction in COPD |
|  | Mucin Hyperproduction in Goblet and Mucous Cells |
|  | Polycystins Mutation Effects in Cystic Kidney Disease |
|  | Plasmin Role in Glomerulonephritis |
|  | Mesangial Cell Dysfunction in Glomerulonephritis |
|  | Interstitial Fibroblasts in Pyelonephritis |
|  | Sertoli Cells Dysfunction Causes Male Infertility |
|  | CD38/CD3 -> JUN/FOS/NF-kB Signaling in T-cell Proliferation |
|  | CD38/CD19 -> JUN/FOS/NF-kB Signaling in B-cell Proliferation |
|  | CD38 -> cADPR/Calcium Signaling |
|  | CD157 -> cADPR/Calcium Signaling |
|  | ANGPT1 -> CREB/CREBBP Expression Targets |
|  | ANGPT2 -> AP-1/CREBBP/MYC Expression Targets |
|  | AREG -> CREB/CREBBP Expression Targets |
|  | AREG -> NCOR2 Expression Targets |
|  | AREG -> SMAD1 Expression Target |
|  | AREG -> HIF1A Expression Targets |
|  | AREG -> AP-1 Expression Targets |
|  | Noradrenaline/Gs Expression Targets |
|  | Noreadrenaline/Gq Expression Targets |
|  | Epinephrine/Gs Expression Targets |
|  | Acetylcholine Expression Targets |
|  | Serotonin/Gq Expression Targets |
|  | Epinephrine/Gq Expression Targets |
|  | Dopamine/Gi Expression Targets |
|  | Aryl Hydrocarbon Receptor Genomic and non-Genomic Signaling |
|  | B-Cell Receptor -> AP-1 Signaling |
|  | BTC -> AP-1/ATF/CREB Expression Targets |
|  | BTC -> EP300/ETS/ETV/SP1 Expression Targets |
|  | SELE -> ELK-SRF Signaling |
|  | CD19 Expression Targets |
|  | CD81 -> AP-1 Expression Targets |
|  | CD247 Expression Targets |
|  | gamma Globulins Expression Targets |
|  | FCGR3A Expression Targets |
|  | CD19/CR2 Expression Targets |
|  | CD72 -> CREB/CREBBP Expression Targets |
|  | CD8 -> ATF/CREB/CREBBP Expression Targets |
|  | CD80 -> NF-kB Expression Targets |
|  | CD86 -> NF-kB Expression Targets |
|  | CXCL6 Expression Targets |
|  | CXCL1 Expression Targets |
|  | CXCL2 Expression Targets |
|  | CXCL3 Expression Targets |
|  | CXCL5 Expression Targets |
|  | Clozapine Induced Granulocytopenia |
|  | EGF -> HIF1A Expression Targets |
|  | EGF -> AP-1/ATF Expression Targets |
|  | EGF -> CREB/CREBBP/ELK/SRF/MYC Expression Targets |
|  | EGF -> TP53 Expression Targets |
|  | EGF -> NCOR2 Expression Target |
|  | EGFR -> NCOR2 Signaling |
|  | EGFR/ERBB2 -> TP53 Signaling |
|  | ERBB2/3 -> EP300/ETS/ETV/SP1 Signaling |
|  | EGFR -> SMAD1 Signaling |
|  | EGFR/ERBB2 -> HIF1A Signaling |
|  | EGFR -> AP-1/ATF2 Signaling |
|  | EGFR -> AP-1/CREB/ELK/SRF/MYC Signaling |
|  | EREG -> CREB Expression Target |
|  | EREG -> EP300/SP1 Expression Targets |
|  | EREG -> HIF1A Expression Target |
|  | EREG -> AP-1/ATF Expression Targets |
|  | Erythropoietin -> AP-1/MYC/CREB Expression Targets |
|  | Erythropoietin -> ELK/SRF Expression Targets |
|  | PECAM1 Expression Targets |
|  | NCAM1 Expression Targets |
|  | F2 -> AP-1/CREB/ELK/SRF/SP1 Expression Targets |
|  | PLG -> AP-1/CREB/ELK/SRF/SP1 Expression Targets |
|  | FGF9 -> AP-1/CREB/MYC Expression Targets |
|  | FGF10 -> AP-1/CREB/CREBBP/MYC Expression Targets |
|  | FGF1 -> AP-1/CREB/ELK/SRF/MYC Expression Targets |
|  | FGF1 -> NCOR2 Expression Target |
|  | FGF7 -> RUNX Expression Targets |
|  | FGF8 -> AP-1/CREB/MYC Expression Targets |
|  | FGF1 -> RUNX Expression Targets |
|  | FGF18 -> AP-1/CREB Expression Targets |
|  | FGF23 -> NCOR2 Expression Targets |
|  | FGF8 -> RUNX Expression Targets |
|  | FGF9 -> RUNX Expression Targets |
|  | FGF2 -> NCOR2 Expression Targets |
|  | FGF4 -> AP-1/MYC Expression Targets |
|  | FGF7 -> AP-1/CREB/CREBBP/MYC Expression Targets |
|  | FGF2 -> RUNX Expression Targets |
|  | FGF2 -> AP-1/CREB/CREBBP/ELK/SRF/MYC Expression Targets |
|  | FGFR -> RUNX2 Signaling |
|  | FGFR -> AP-1/CREB/CREBBP/ELK/SRF/MYC Signaling |
|  | ADCYAP1 Expression Targets |
|  | FSHR Expression Targets |
|  | Glutamate/Gq Expression Targets |
|  | VIP Expression Targets |
|  | GH1/GHR -> NF-kB/ELK/SRF/MYC Expression Targets |
|  | CSH1/GHR Expression Targets |
|  | HBEGF -> AP-1/ATF Expression Targets |
|  | HBEGF -> TP53 Expression Targets |
|  | HBEGF -> EP300/ETS/ETV/SP1 Expression Targets |
|  | HBEGF -> HIF1A Expression Targets |
|  | HBEGF -> CREB/MYC Expression Targets |
|  | HGF -> AP-1/CREB/ELK/SRF/MYC Expression Targets |
|  | HGFR -> AP-1/CREB/MYC Signaling |
|  | TAM Receptor Signaling Activation in Cancer |
|  | G0/G1 Cell Cycle Phase Transition Activation in Cancer |
|  | TGFB Signaling activation by Blocking of Tumor Suppressors |
|  | ANGPT/TEK Stimulates Endothelial Cell Migration in Cancer |
|  | VEGF Independent Angiogenesis in Cancer |
|  | Integrins in Cancer Cell Motility, Invasion and Survival |
|  | CDH2 Activation Promotes Cancer Cell Migration and Survival |
|  | TGFB Family in Epithelial to Mesenchymal Transition in Cancer |
|  | Treg Cells Promote Immunosuppression in Cancer Immune Escape |
|  | IGF2 -> HIF1A/MYC Expression Targets |
|  | IGF1 -> ELK/SRF/HIF1A/MYC/SREBF Expression Targets |
|  | Insulin -> ELK/SRF/HIF1A/MYC/SREBF Expression Targets |
|  | NCAM1 -> CREB/ELK/SRF/MYC Signaling |
|  | CD19 -> JUN/ELK Signaling |
|  | PECAM -> SP1 Signaling |
|  | FcIgER -> ELK-SRF Signaling |
|  | ICAM1 -> AP-1/CREB/ELK/SRF Signaling |
|  | IGF1R -> ELK/SRF/HIF1A/MYC/SREBF Signaling |
|  | InsulinR -> ELK/SRF/SREBF Signaling |
|  | FibronectinR -> AP-1/ELK/SRF/SREBF Signaling |
|  | Fibronectin Expression Targets |
|  | CTGF -> AP-1/CREB/MYC Expression Targets |
|  | CTGF -> NCOR2 Expression Target |
|  | ICAM1 Expression Targets |
|  | IL6R -> CEBP/ELK/SRF Signaling |
|  | IL2R -> ELK/SRF/MYC Signaling |
|  | IL4R -> ELK/SRF/HMGY Signaling |
|  | IL16 -> AP-1 Expression Targets |
|  | IL6 Expression Targets |
|  | IL4 Expression Targets |
|  | IL2 Expression Targets |
|  | UrokinaseR -> ELK/SRF Signaling |
|  | PTPRC -> BCL6 Expression Targets |
|  | Leptin -> ELK/SRF Expression Targets |
|  | Prostaglandin F Expression Targets |
|  | Thromboxane A2 Expression Targets |
|  | LPA Expression Targets |
|  | PAF/Gq -> AP-1/ATF1/CREB/ERK/SRF Expression Targets |
|  | Dronabinol/Anandamide Expression Targets |
|  | Morphine Expression Targets |
|  | PAF/Gq -> NF-kB Expression Targets |
|  | S1P Expression Targets |
|  | PGE1 Expression Targets |
|  | NRG1 -> EP300/ETS/ETV/SP1 Expression Targets |
|  | NRG1 -> CREB/CREBBP/ELK/SRF/MYC Expression Targets |
|  | NRG1 -> TP53 Expression Target |
|  | NRG1 -> AP-1/ATF Expression Targets |
|  | NRG1 -> HIF1A Expression Target |
|  | NGF -> SMAD3/NF-kB Expression Targets |
|  | NGF -> AP-1/TP53/MYC Expression Targets |
|  | NGF -> FOXO/MYCN/ELK-SRF Expression Targets |
|  | BDNF Expression Targets |
|  | NTF3 Expression Targets |
|  | NTF4 Expression Targets |
|  | NGF -> CREB/CEBPB/MEF2A Expression Targets |
|  | Adenosine Expression Targets |
|  | PDGFB -> AP-1/CREB/MYC Expression Target |
|  | PDGFC -> CREB Expression Target |
|  | PDGFD -> AP-1 Expression Targets |
|  | PDGF -> AP-1/CREB/CREBBP/MYC Expression Targets |
|  | KIT -> MITF Signaling |
|  | PDGFR -> AP-1/MYC Signaling |
|  | AVP/Gs -> CREB/ELK/SRF/AP-1/EGR Expression Targets |
|  | GNRH1 Expression Targets |
|  | NTS Expression Targets |
|  | GAST Expression Targets in GAST Expression Targets |
|  | AGT -> TP53 Expression Targets |
|  | GNRH2 Expression Targets |
|  | OXT Expression Targets |
|  | POMC Expression Targets |
|  | CCL11 Expression Targets |
|  | EDN1 Expression Targets |
|  | AGT -> CREB Expression Targets |
|  | NPY Expression Targets |
|  | IFNA1/Gq Expression Targets |
|  | AGT -> ELK/SRF Expression Targets |
|  | TAC1 Expression Targets |
|  | EDN3 Expression Targets |
|  | AVP/Gq -> CREB/ELK/SRF/AP-1/EGR Expression Targets |
|  | PRL/GHR -> NF/kB/ELK/SRF/MYC Expression Targets |
|  | PDCD1 -> AP-1 Expression Targets |
|  | PTPRC -> BCL6 Signaling |
|  | AngiopoietinR -> AP-1 Signaling |
|  | GDNF -> HSF1 Signaling |
|  | NTRK -> AP-1/CREB/ELK/SRF/MYC/SMAD3/TP53 Signaling |
|  | GAS6 -> AP-1/CREB Expression Targets |
|  | FIGF -> AP-1 Expression Target |
|  | FIGF -> NCOR2 Expression Target |
|  | KITLG -> AP-1/CREB/CREBBP/MYC Expression Targets |
|  | GDNF -> HSF1 Expression Targets |
|  | CSF1 -> AP-1/CREB/CREBBP/MYC Expression Targets |
|  | PGF -> AP-1/CREB/CREBBP/MYC Expression Targets |
|  | FLT3LG -> AP-1/CREB/CREBBP Expression Targets |
|  | Lysophosphatidic Acid/LPARs Signaling |
|  | Ras-GRF Regulation Signaling |
|  | Androgen Receptor non-Genomic Signaling |
|  | MERTK Signaling |
|  | Ras Signaling |
|  | TGF-beta Signaling |
|  | AMPK Signaling |
|  | Ras-GAP Regulation Signaling |
|  | AXL Receptor Tyrosine Kinase Signaling |
|  | PI3K/RAC1 Signaling |
|  | VEGF Signaling |
|  | EGFR Signaling |
|  | ERK/MAPK Canonical Signaling |
|  | TCR -> AP-1 Expression Targets |
|  | T-Cell Receptor -> AP-1 Signaling |
|  | TGFA -> TP53 Expression Targets |
|  | TGFA -> HIF1A Expression Targets |
|  | TGFA/AP-1/ATF Expression Targets |
|  | TGFA -> CREB/CREBBP/ELK-SRF/MYC Expression Targets |
|  | LTA Expression Targets |
|  | TNF -> AP-1 Expression Targets |
|  | TNF -> ELK-SRF Expression Targets |
|  | TNF -> CREB Expression Targets |
|  | TNF -> TP53/ATF Expression Targets |
|  | TNFRSF1A -> AP-1/ATF/TP53 Signaling |
|  | TNFRSF1A -> CREB/ELK-SRF Signaling |
|  | Thrombopoietin -> SPI1 Expression Targets |
|  | Thrombopoietin -> AP-1/CREB/CREBBP/MYC Expression Targets |
|  | Thrombopoietin -> SP1 Expression Targets |
|  | IL1B -> NO Expression Targets |
|  | ROS in Triggering Vascular Inflammation |
|  | ROS and RNS in Vasoconstriction and Vasodilation Regulation |
|  | ROS in Angiotensin Mediated Cardiovascular Remodeling and Hypertrophy |
|  | PLAU -> ELK-SRF/AP-1 Expression Targets |
|  | VEGFA -> ATF/CREB/ELK-SRF Expression Targets |
|  | VEGFA -> NFATC Expression Targets |
|  | VEGFC -> ATF Expression Target |
|  | VEGFA -> AP-1/CREBBP/MYC Expression Targets |
|  | VEGFA -> NCOR2 Expression Target |
|  | VEGFR -> NFATC Signaling |
|  | VEGFR -> AP-1/CREB/MYC Signaling |
|  | VEGFR -> ATF/CREB/ELK-SRF Signaling |
|  | pathway 3 |
|  | ace2 |
|  | prarg negative regulators, ps-positive |
|  | Neighbors of Ras GTPase |
|  | Model of PPARG signaling in psoriasis |
|  | PPARG negative regulators and targets |
|  | Model of PPARG related pathways in psoriasis (short version) |
|  | non-proteinal partners of SARSs |
|  | H2A-K120, H2A-K119 and H3-K4 |
|  | Epigenetic Modifications of H2A-K120, H2A-K119 and H3-K4 |
|  | 1\_Inflammation in keratinocytes in the pathology of acne |
|  | 2\_Hyperseborrhea in acne vulgaris |
|  | 2\_Acute phase initiation in atopic dermatitis |
|  | 2\_Interleukin-17 and interleukin-22 signaling in psoriasis |
|  | 1\_The onset of atopic dermatitis |
|  | 1\_TNF and IL1B provoke ECM degradation in osteoarthritis |
|  | 2\_Osteoblast-mediated bone demineralization |
|  | 3\_The role of interstitial fibroblasts in pyelonephritis |
|  | 3\_Enhanced angiogenesis in endometriosis |
|  | 1\_Disruption of luteinizing hormone and follicle-stimulating hormone secretion in PCOS |
|  | 2\_Cardiomyocyte hypertrophy |
|  | 3\_Dysfunction of smooth muscle cells in hypertension |
|  | 1\_Rise in aldosterone synthesis in adrenal zona glomerulosa cells |
|  | 2\_The intense proliferation and contractile activity of smooth muscle cells in pulmonary hypertension |
|  | 1\_Cigarette smoke causes dysfunction and production of inflammatory mediators in alveolar epithelial cells |
|  | 3\_Role of alveolar macrophages in pulmonary tissue destruction in COPD |
|  | 1\_Th2 cell response in asthma |
|  | 3\_1\_Mucus accumulation in asthma |
|  | 2\_2\_Eosinophilia and neutrophilia in asthma: eosinophil apoptosis |
|  | 4\_Mucin hyperproduction in goblet and mucous cells in COPD |
|  | 1\_CD4+ T cell and epithelial cell response in celiac disease |
|  | 2\_CD8+ T-cell response and mucosal damage in celiac disease |
|  | 1\_2\_Insulin synthesis and secretion: insulin secretion |
|  | 1\_1\_Insulin synthesis and secretion: insulin processing and storage |
|  | 4\_1\_Insulin resistance: insulin signaling |
|  | 3\_2\_Beta-cell proliferation and compensation: neogenesis/dedifferentiation |
|  | 3\_Islet beta-cell destruction in T1D |
|  | 2\_1\_General pathways of thyroid hormone signaling |
|  | New Pathway |
|  | HRAS->RAF->MAPK1/3 |
|  | KRAS->RAFs->MAPK1/3 |
|  | NRAS->RAFs->MAPK1/3 |
|  | Other RASs-RAFs-MAPK1/3 |
|  | Interleukin-17 and interleukin-22 signaling in psoriasis |

---

|  |  |
| --- | --- |
| Group | non protein partners |

---

|  |  |
| --- | --- |
| Source | curated |

---

|  |  |
| --- | --- |
| MedScan ID | 12103930 |

---

|  |  |
| --- | --- |
| Alias | Ras GTP binding proteins |
|  | Ras guanosine triphosphatase |
|  | Ras small monomeric GTPases |
|  | Ras guanosine triphosphatases |
|  | Ras-related small GTPases |
|  | Ras oncogene homolog |
|  | Ras activity |
|  | Ras GTPases |
|  | Ras-related small GTPase |
|  | Ras GTP binding protein |
|  | Ras small monomeric GTPase |
|  | Ras activities |
|  | Ras GTPase |
|  | Ras |
|  | Ras small GTPases |
|  | Ras small GTPase |
|  | small GTPase Ras |
|  | Ras protein |
|  | Ras proteins |
|  | small GTPases Ras |
|  | Ras small monomeric GTPase activity |
|  | (RAS) |
|  | Ras oncogene homologs |
|  | Ras family small GTPases |
|  | ARABIDOPSIS THALIANA SECRETION-ASSOCIATED RAS SUPER FAMILY |
|  | Ras family of GTPases |
|  | Ras-GTPase |
|  | SECRETION-ASSOCIATED RAS |

---

|  |  |
| --- | --- |
| Cell Localization | Cytoplasm |

---

|  |  |
| --- | --- |
| LocatorString | pmquery:curcumin prostate cancer |

---
